# Peri-tumoral brain edema associated with glioblastoma correlates with tumor recurrence

**DOI:** 10.7150/jca.53198

**Published:** 2021-02-05

**Authors:** Xingping Qin, Rui Liu, Farhana Akter, Lingxia Qin, Qiurong Xie, Yanfei Li, Haowen Qiao, Wen Zhao, Zhihong Jian, Renzhong Liu, Songlin Wu

**Affiliations:** 1 Department of Neurosurgery, Renmin Hospital of Wuhan University, Wuhan, Hubei 430060, China.; 2 John B. Little Center for Radiation Sciences, Department of Environmental Health, Harvard T.H. Chan School of Public Health, Boston, MA 02115, USA.; 3 Department of Physiology, School of Basic Medical Sciences, School of Medicine, Wuhan University, Wuhan, Hubei 430071, China.; 4 Faculty of Arts and Sciences, Harvard University, Cambridge, MA 02138, USA.; 5 Department of Neurology, Renmin Hospital of Wuhan University, Wuhan, Hubei 430060, China.; 6 Department of Gynecology and Obstetrics, Renmin Hospital of Wuhan University, Wuhan, Hubei 430060, China.; 7 Department of Orthopedics, The First Affiliated Hospital of Jinan University, Guangzhou 510630, China.; 8 Department of Biomedical Engineering, Wuhan University School of Basic Medical Sciences, Wuhan, Hubei 430071, China.; 9 Department of Geriatrics, Renmin Hospital of Wuhan University, Wuhan, Hubei 430060, China.

**Keywords:** Glioblastoma, Peritumoral brain edema, Recurrence, Tumor resection.

## Abstract

Glioblastoma is the most common malignant tumor of the brain. Despite advances in treatment, the prognosis for the condition has remained poor. Glioblastoma is often associated with peritumoral brain edema (PTBE), which can result in increased intracranial pressure and devastating neurological sequelae if left untreated. Surgery is the main treatment for glioblastoma, however current international surgical guidelines do not specify whether glioblastoma-induced PTBE tissue should be resected. In this study, we analyzed treatment outcomes of PTBE using surgical resection. We performed a retrospective analysis of 255 cases of glioblastoma between 2014 and 2016, and found that a significant proportion of patients had a degree of PTBE. We found that surgical resection led to reduction in midline shift that had resulted from edema, however, postoperative complications and KPS scores were not significantly different in the two conditions. We also observed a delay in glioblastoma recurrence in patients undergoing PTBE tissue resection vs patients without resection of PTBE tissue. Interestingly, there was an abnormal expression of tumor associated genes in PTBE, which has not been previously been found. Taken together, this study indicates that glioblastoma-induced PTBE should be investigated further particularly as the tumor microenvironment is a known therapeutic target and therefore interactions between the microenvironment and PTBE should be explored. This study also highlights the importance of resection of PTBE tissue to not only reduce the mechanical obstruction associated with edema but also to delay recurrence of glioblastoma.

## Introduction

Gliomas are a heterogenous group of brain tumors. The World Health Organization (WHO) classification system classifies gliomas from grade 1 to grade 4. Grade 1 and 2 gliomas are referred to as low-grade gliomas (LGG), while grade 3 and 4 gliomas are referred to as high-grade gliomas (HGG) 1. Glioblastoma (GBM) is the most malignant form and is usually treated with surgical resection. However, the prognosis for GBM remains poor, with an average survival time of 14 months post treatment 2. The preoperative evaluation of GBMs relies on magnetic resonance imaging (MRI), which has the highest sensitivity to tumors 3. The standard surgical approach is to maximally resect tumors whilst preserving neurological function. However, GBM is associated with high relapse rates and therefore has an unfavorable prognosis.

Tumors are often accompanied by abnormal expression of tumor-associated genes. The AKT gene, also known as protein kinase B plays an important role in cell survival and activation of AKT promotes cell proliferation 4,5,6. The extracellular signal-regulated kinase (ERK) signaling pathway is at the core of signaling networks that regulate cell growth, development, and division 7,8. There are several tumor suppressor genes that can be affected in tumorigenesis. Phosphatase and tension homologue deleted on chromosome 10 (PTEN) and P53 are tumor suppressors that are frequently disrupted during carcinogenesis. Their expression is maintained in normal cells but are significantly down-regulated during carcinogenesis 9,10.

**Figure 1:**
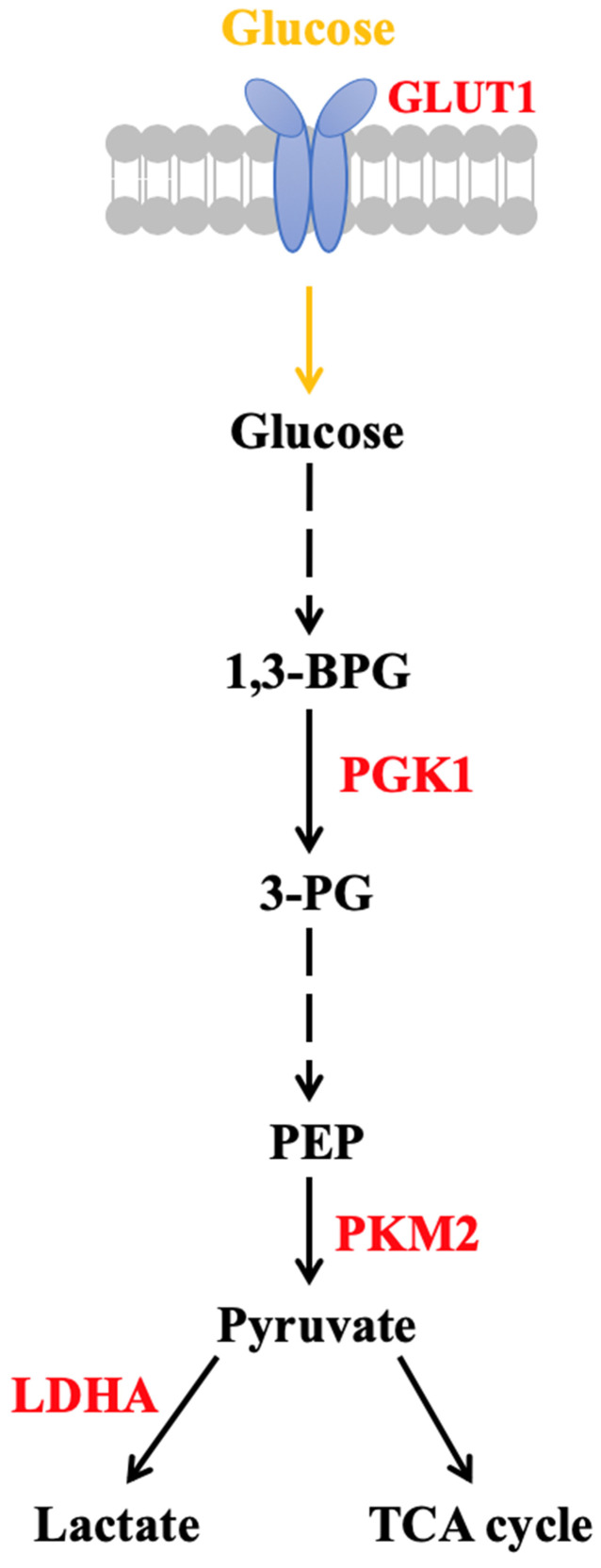
Warburg effect and tumor growth

Previous studies have indicated that the metabolic circuit of glioma cells changes during carcinogenesis, with evidence of an increase in glucose uptake and lactic acid production regardless of the availability of oxygen 11. We call this phenomenon aerobic glycolysis or Warburg effect, and this is closely related to tumor growth 12. Direct regulators of the Warburg effect include Pyruvate kinase M (PKM2), phosphoglycerate kinase 1 (PGK1), lactate dehydrogenase A (LDHA), c-Myc oncogene and Glucose transporter 1 (GLUT1) 13,14. An increase in PKM2 expression level in tumors further promotes LDHA, c-Myc and GLUT1 expression 15. PGK1 promotes the Warburg effect in tumors by suppressing tricarboxylic acid cycle (TCA) through mitochondrial translocation 13,14,16,17 (Fig. 1). Peritumoral brain edema (PTBE) is commonly found in GBM and is thought to be associated with poorer prognosis 18 and increased morbidity such as cognition deficits 19. PTBE can also be found in metastatic brain tumors and benign tumors such as meningiomas 20.

There is controversy regarding whether PTBE associated with GBM should be resected. In this study we demonstrate that surgical resection of PTBE tissue does not confer a greater post-operative risk compared to patients who do not receive surgical resection. Most interestingly, we demonstrate that GBM associated PTBE is associated with an upregulation of tumor-associated genes, which may be involved in the pathogenesis of GBM formation and evolution and should be explored further.

## Materials and Methods

### Peritumoral brain edema (PTBE)

The maximum diameter of the tumor was measured on T1 weighted images (T1WI), and the maximum diameter of the edema band was measured axially on the T2-weighted images (T2WI) or Fluid-attenuated inversion recovery (FLAIR) sequence images. PTBE was evaluated for the maximum diameter of the edema and was classified into 3 grades: Level 0, no edema; Level 1, maximum diameter less than 1cm; Level 2, maximum diameter >1cm. Axial, coronal and sagittal MRI scans were reviewed in all cases and the maximum extent of PTBE was measured using these scans.

### Neurosurgery

We divided patients with PTBE into two groups: one group received surgical resection of the tumor and PTBE tissue and the other group received surgical resection of tumor only. In this study, 50 males (51%) and 35 females (53.8%) received surgical resection of PTBE tissue in addition to tumor resection, while 48 males (49%) and 30 females (46.2%) received surgical excision of tumor only. We also categorized patients according to age as young (<60 years) and elderly (≥60 years). 53 patients (55.8%) in the younger group and 32 patients (47.1%) in the elderly group received both PTBE and tumor resection, whilst 42 (44.2%) young patients and 36 (52.9%) elderly patients received surgical excision of the tumor only.

We used T2WI and T2-FLAIR MRI images and diffusion tensor imaging (DTI) as a reference during surgery. All operations were conducted with a microscope. For the tumor only group, we resected the tumor completely and ensured that the surrounding normal brain tissue was preserved. We resected the PTBE tissue according to T2W1 and T2-FLAIR images, aiming to preserve as much of the eloquent brain areas as possible without damaging the nerve fiber tracts. Patients were subjected to intraoperative neurophysiological monitoring under awake conditions to identify any neurological impairment. Some patients underwent chemotherapy postoperatively, while some underwent radiotherapy, and some received combination of chemotherapy and radiotherapy. All cases were categorized according to their pathology, in the "tumor only" group, 35 patients (44.80%) were classified as of the MGMT methylated type, 70 patients (89.70%) as IDH1 mutated, 40 patients (51.30%) as EGFR amplified, 42 patients (53.80%) as PI3K activated, 4 patients (5.12%) with 1p/19q codeletion, 19 patients (24.35%) with P53 mutations, and 42 patients (53.85%) with PTEN mutations. In the "tumor only + PTBE" group, there are 38 patients (44.71%) who had MGMT methylation, 77 patients (90.59%) with IDH1 mutation, 45 patients (52.94%) with EGFR amplification, 43 patients (50.59%) with PI3K activation, 6 patients (7.06%) with 1p/19q codeletion, 22 patients (25.88%) with P53 mutation, and 48 patients (56.47%) with PTEN mutation.

### Human PTBE brain tissues

Human PTBE brain tissues were obtained at the time of surgery from Department of Neurosurgery in Renmin Hospital of Wuhan University, and histological diagnosis was confirmed independently by three neuropathologists. The procurement of tissue usage and all image data for the study was obtained with written patient-informed consent and approved by the Institutional Ethics Committee Faculty of Medicine at Renmin Hospital of Wuhan University (approval number: 2012LKSZ(010)H).

### Normal human brain tissues

Normal brain tissue was obtained from donors who had no known diseases or pathology at post-mortem via the Chinese Brain Bank Center (CBBC) through a human body donation program, organized by the Wuhan Red Cross Society. Specific permission for the use of autopsy results and medical data for research purposes were obtained either from the donors themselves or from their relatives, and was also approved by the Biomedical Research Ethics Committee of South-Central University for Nationalities (approval number: 2017-SCUEC-MEC-004).

### KPS

The patients were assessed by Karnofsky performance scale (KPS). This is an assessment tool to identify functional impairment when comparing effectiveness of different therapies. The score is calculated from 0 to 100, with 0 representing death and 100 representing no signs or symptoms of disease. A score ≥70 signifies that one is able to care for themselves independently.

### Midline shift

Midline shift is a measure of the brain past its center line and indicates a mass effect from the presence lesions such as a tumor or edema. Midline shift is a common characteristic of GBM. The presence of midline shift is considered as an independent prognostic factor influencing survival among GBM patients 21. In this study, we measured the distance between septum pellucidum from the midline using MRI and is classified into 2 groups: <1 cm and >1cm.

### Western blotting analysis

Western blotting was performed using a standard protocol. Briefly, the polyvinylidene difluoride (PVDF) membrane by Millipore (USA) was used to incubate with the first antibody against AKT (Mouse, 1:1000), phospho-AKT (Ser473) (Rabbit, 1:1000), ERK 1/2 (Rabbit, 1:2000), phospho-ERK 1/2 (Thr^202^/Tyr^204^) (Rabbit, 1:2000), PTEN (Rabbit, 1:1000), P53 (Mouse, 1:1000), PKM2 (Rabbit, 1:1000), LDHA (Rabbit, 1:1000), c-Myc (Rabbit, 1:1000), Actin (Rabbit, 1:2000) (#8457) from Cell Signaling Technology (MA, USA), PGK1 (Rabbit, 1:2000), GLUT1 (Rabbit, 1:1000), GAPDH (Mouse, 1:5000) from Proteintech Group (USA), NLGN3 (Mouse, 1:500) (sc-137052) that was from Santa Cruz Biotechnology. The primary antibody was labeled with the secondary antibody and the protein bands were imaged using SuperSignal West Femto Maximum Sensitivity Substrate (Pierce, Rockford, IL, USA). Blot images were obtained directly from the PVDF membrane using an EC3 imaging system (UVP, LLC, Uplant, USA). Western blot data was quantified using Image J Pro Plus 6.0 software.

### Statistics

All data are expressed as mean ± SE. Newman-Keuls tests were used for post-hoc comparisons when appropriate. Chi-Square tests were used to compare KPS scores and midline shift between resected and non-resected cases. The Fisher Exact test was used to compare recurrence rates following resection of PTBE vs patients who did not receive resection. *P<*0.05 was considered statistically significant.

## Results

### Glioblastoma induces peritumoral brain edema

**Figure 2:**
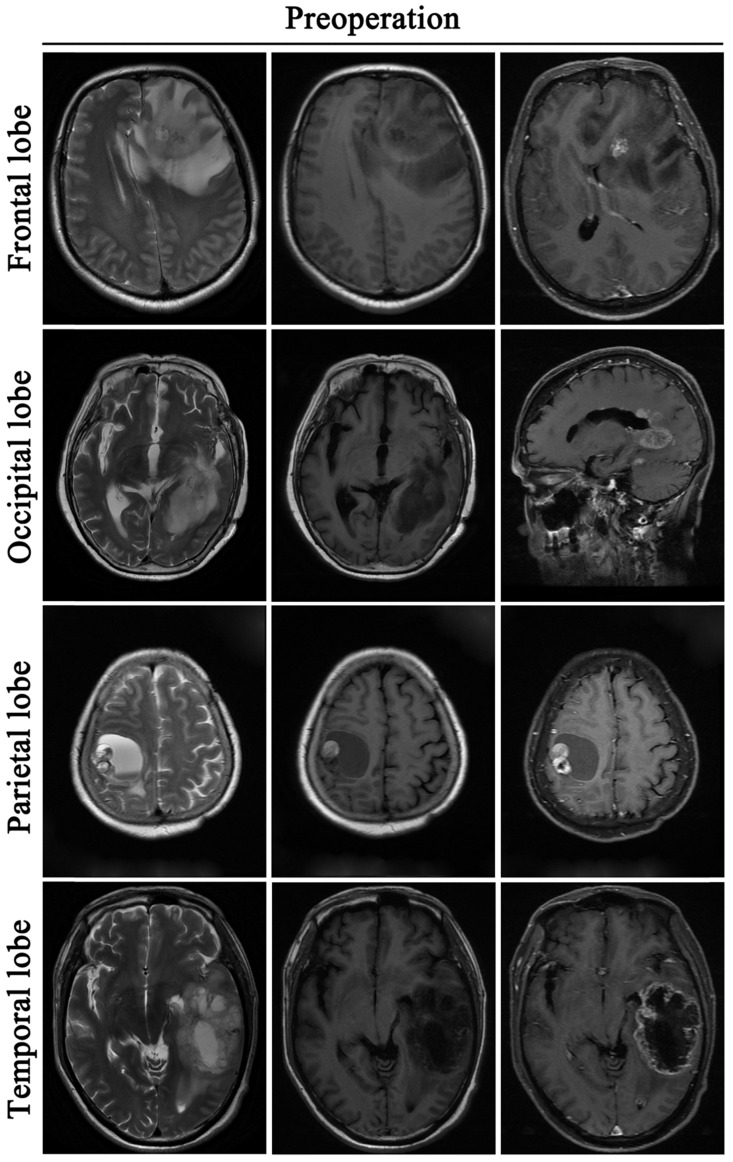
** Glioblastoma induces peritumoral brain edema.** All the tumors are located in the cortex: Frontal lobe, Occipital lobe, Parietal lobe, Temporal lobe. Before the operative, these patients are accompanied by peritumoral brain edema.

A retrospective study was performed on 255 GBM patients from 2014 to 2016, who underwent tumor resection. Among them, 207 patients were followed up for more than 1 year and 48 patients were not followed up due to death or unknown reasons. Demographic characteristics of patients are specified in Table 1. Data shows that the median age in this cohort is 52 years. In the 207 patients who were followed up, 55.07% (114/207) were men and 44.93% (93/207) were women. The primary site of GBM occurrence was in the frontal lobe (84 cases), followed by the temporal lobe (67 cases), 35 cases in the parietal lobe, and 21 cases in the occipital lobe. PTBE was graded into 3 grades as follows: Level 0 (no edema); Level 1 (maximum diameter of edema less than 1cm); Level 2 (maximum diameter of edema >1cm). We found that brain edema was present in 78.74% (163/207) of the cases of GBM) (Fig. 2). There were 92 cases graded as level 0, 36 cases graded as level 1 and 127 cases graded as level 2.

### Management of PTBE is based on the clinical condition of the patient

**Figure 3:**
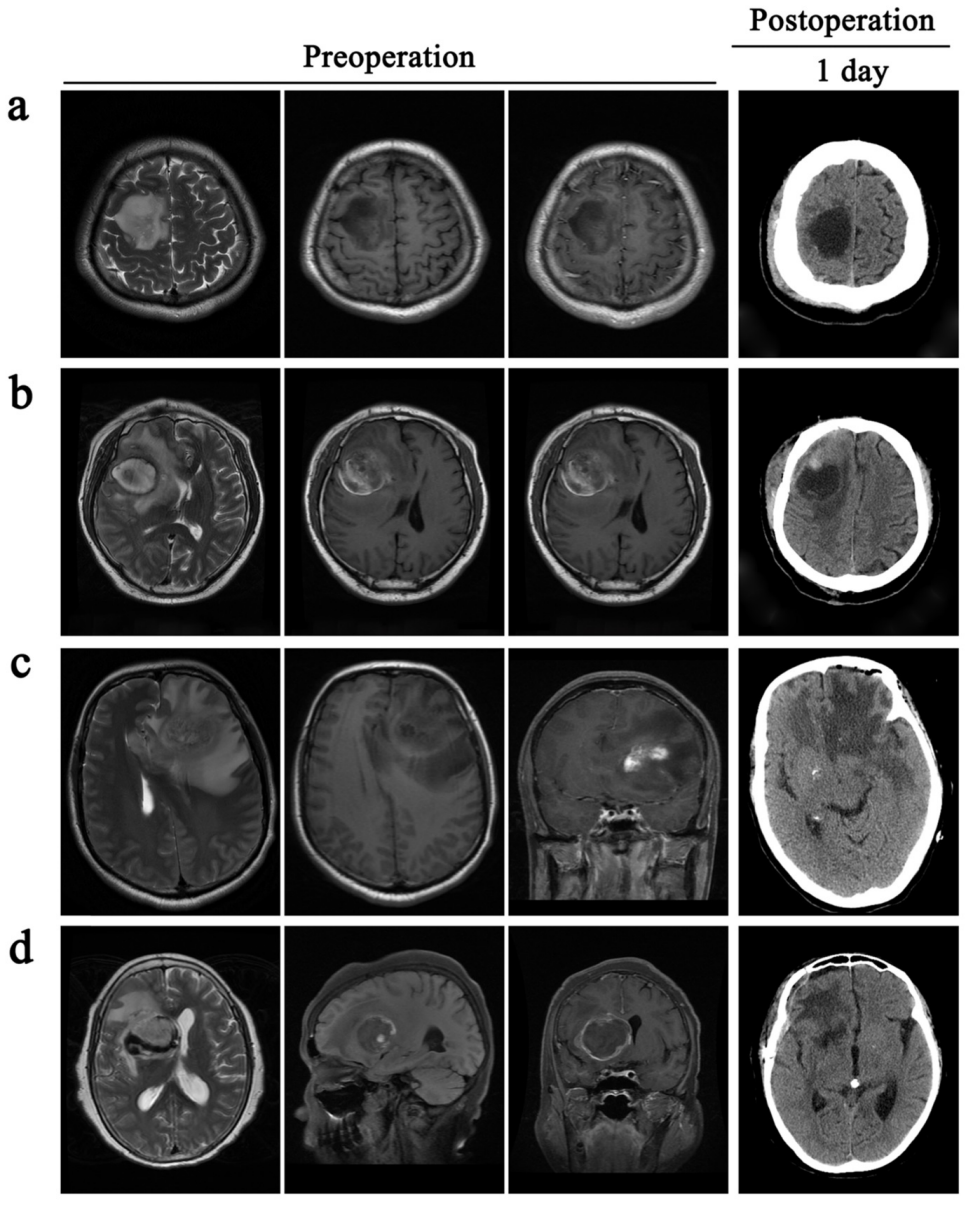
** Surgical resection of PTBE is clinically based on the condition of patients.** All the patients are accompanied by peritumoral brain edema before operative. a and c, patients only take a resection of the tumor. b and d, patients remove the tumor and give an excision with PTBE.

All patients received total excision of their tumors. However, the decision to surgically resect PTBE tissue based on the extent of PTBE. In total, 85 patients received surgical resection of PTBE tissue in addition to tumor resection and 78 patients received surgical excision of their tumor only (Table 2). In one example case of a patient with right frontal lobe tumor in Fig. 3a, there was little evidence of PTBE and therefore we decided to resect the tumor only. In Fig. 3b, the patient presented with a right frontal lobe tumor, with contrast enhancement in T1WI-enhanced images and PTBE grading of level 1. We therefore resected both tumor and PTBE tissue. In **Fig. 3c**, the patient presented with a tumor in the left frontal lobe, with contrast enhancement and PTBE level 1. This patient received surgical resection of tumor and PTBE tissue. In **Fig. 3d**, the patient presented with a tumor in the right frontal lobe, with contrast enhancement and PTBE level 2; this patient therefore had surgical excision and removal of PTBE tissue.

### Postoperative complications and KPS scores do not show significant differences in patients with PTBE excision or no resection

Neurological complications following surgery include headache, vomiting, paresthesia, hemiplegia, visual impairment, aphasia and seizures. In Table 3, we recorded in detail the number of patients with corresponding postoperative complications at 1 month, 3 months, and 6 months after surgery.

In patients with headache, PTBE tissue resection accounted for 42.75% of cases, compared to 43.16% for cases without resection. In patients with vomiting, PTBE tissue resection accounted for 12.55% cases vs 17.52% patients who did not receive resection. In patients with paresthesia, PTBE resection accounted for 34.51% cases, vs 39.32% for patients who did not receive resection. In patients with hemiplegia, PTBE resection accounted for 38.43%, vs 44.4% of patients without resection. In patients with visual impairment, PTBE resection accounted for 9.8% cases vs 14.53% for those without resection. In patients with aphasia, PTBE resection accounted for 10.59%, vs 12.82% in patients without resection. In patients with seizures, PTBE resection accounted for 5.13% cases, vs 6.41% in patients without resection. Together, we did not observe a significant difference in the postoperative complications between resected cases vs those that did not resection of PTBE.

We performed KPS scores on all GBM patients after tumor resection to evaluate the prognosis. In 163 GBM patients with PTBE, KPS scores were above 70 points (≥70, 91.41%; <70, 8.59%). In all patients with a KPS score greater than 70, PTBE tissue resection accounted for 92.55%, vs 94.02% in patients who did not receive resection of PTBE tissue. In all patients with a KPS score less than 70, PTBE tissue resection accounted for 7.45%, vs 5.98% in those without resection. These data suggest that patients do not show significant differences in KPS scores after resection of PTBE tissue compared to patients who did not receive resection.

### PTBE resection eases midline shift in GBM patients

Midline shift is a relatively common phenomenon in GBM patients. In Table 3, statistical analysis revealed significantly improved midline shift in 163 patients undergoing PTBE tissue resection. In 163 GBM patients with PTBE, midline shift pre-operatively was greater than 1cm in the majority of the cases (>1cm, 61.35%; <1 cm, 38.65%). PTBE tissue resection accounted for 68.24% of cases with midline shift less than 1cm post-operatively, vs 53.42% in patients who did not receive resection, In all patients with a midline shift >1cm, PTBE resection accounted for 31.76%.vs. 46.58% in patients who did not receive resection. This suggests that patients show significant differences in midline shift after resection of PTBE tissue compared to those who did not receive resection at 1 month and 6 months after surgery, however, there was no significant difference at 3 months after surgery.

### GBM recurrence in patients undergoing PTBE resection is slower than that of PTBE without resection

**Figure 4:**
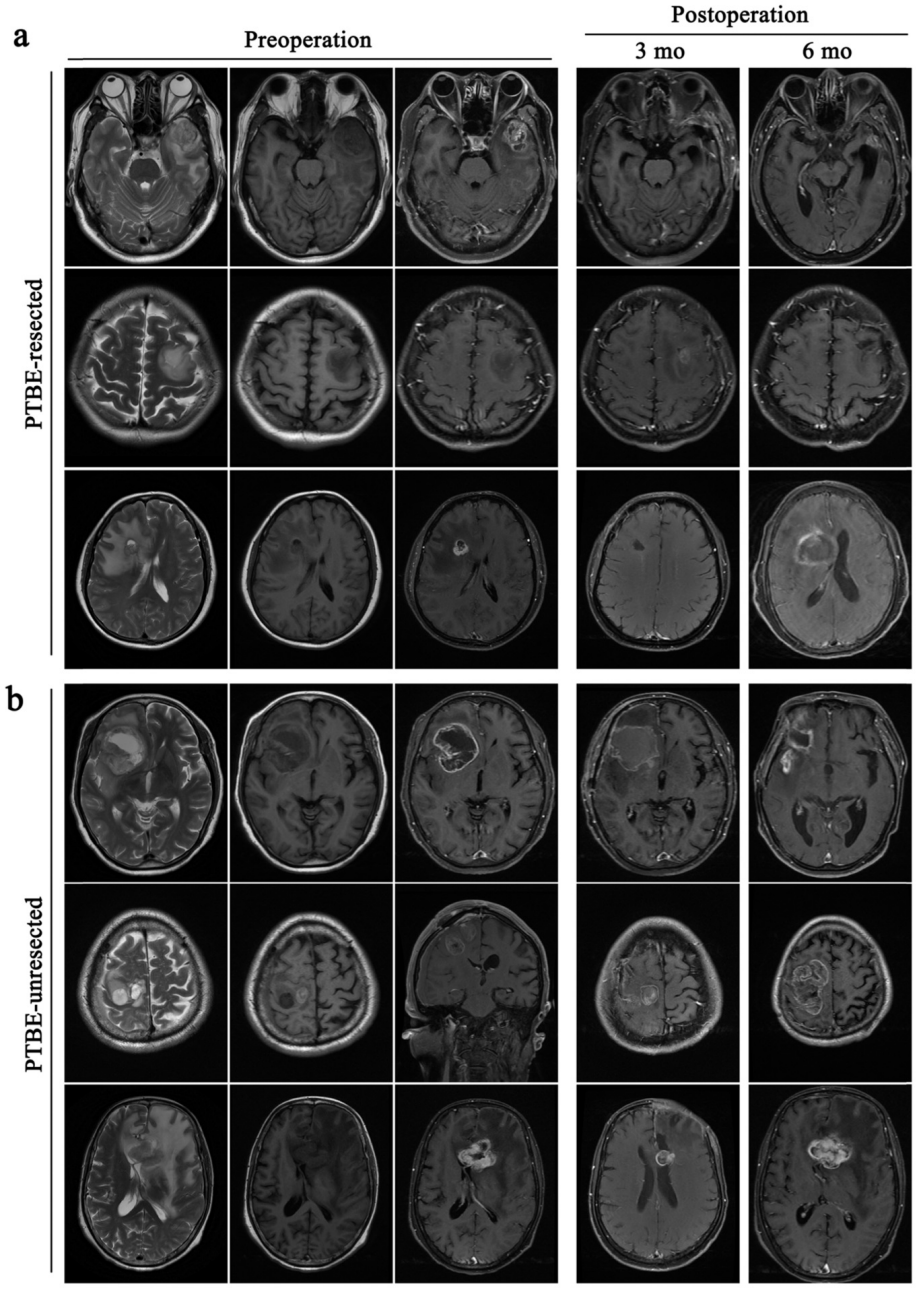
** GBM recurrence in patients undergoing PTBE resection is slower than that of PTBE without resection.** All the patients are accompanied by peritumoral brain edema before operative. a, patients remove the tumor and give an excision with PTBE, the tumor recurred during the 6^rd^ month after operative. b, patients only take a resection of the tumor, the tumor recurred during the 3^rd^ month after operative.

GBM is the most aggressive tumor in the brain with high rates of recurrence even following complete surgical resection. In this study, 207 patients underwent total tumor resection, however they all relapsed within 12 months after operation. In 163 GBM patients with PTBE, the recurrence time of patients who also received resection of PTBE tissue was delayed compared to patients without PTBE tissue (Table 3 and Fig. 4). In patients with both tumor and PTBE tissue resection, there was no recurrence at 1 month's follow-up (0%); 10 patients relapsed at 3 months' follow-up (11.77%), and 74 patients relapsed at 6 months' follow-up (87.06%). In the group with surgical excision of tumor only, 2 patients relapsed at 1 month's follow-up (2.56%), 28 patients relapsed at 3 months' follow-up (35.90%), and 48 patients relapsed at 6 months' follow-up (61.54%).

### Abnormal expression of tumor-associated proteins in PTBE region

**Figure 5:**
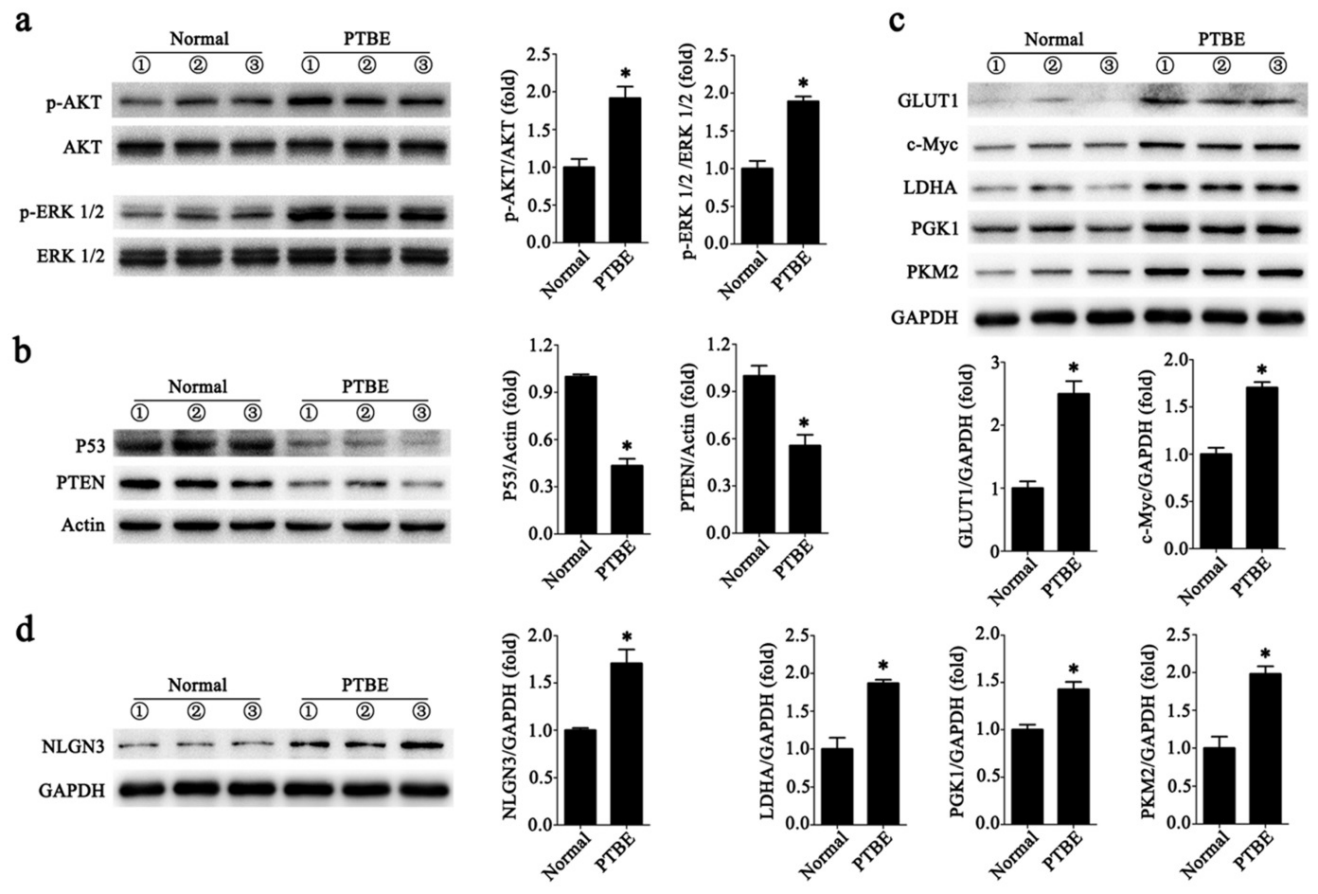
** Abnormal expression of tumor-associated proteins in PTBE region.** a-d, Western blotting analysis of p-AKT and p-ERK 1/2 (a); P53 and PTEN (b); GLUT1, c-Myc, LDHA, PGK1and PKM2 (c); NLGN3 (d) in human brain tissues (*P < 0.05 versus the Normal). Data are expressed as mean ± SE. Statistical analysis was implemented by student's t-test and variance analysis.

We collected tissues from patients with PTBE and normal human brain tissues. In Fig. 5a we show that levels of phosphorylated AKT (p-AKT) and the phosphorylated of ERK 1/2 (p-ERK 1/2) were higher in brain tissues with PTBE compared to control. We also tested the levels of tumor suppressor genes PTEN and P53, and found reduced expression of these in PTBE brain tissues compared to normal brain tissues (Fig. 5b). Previous studies have demonstrated that PKM2, PGK1, LDHA, c-Myc and GLUT1 plays a crucial role in tumorigenesis. Western blot results showed that PKM2, PGK1, LDHA, c-Myc and GLUT1 expression in PTBE brain tissue were up-regulated compared to normal brain tissue (Fig. 5c). In addition, recent studies have shown that NLGN3 plays an important role in tumor growth 4,22. Our recent report shows that NLGN3 levels are associated with postoperative recurrence of gliomas 5, western blotting analysis of NLGN3 showed that the level of NLGN3 was higher in the PTBE region compared to normal brain tissue (Fig. 5d). The results suggest that increased expression of tumor-associated proteins in the PTBE region may be associated with relatively rapid recurrence of tumors.

## Discussion

Glioblastoma is the most aggressive tumor in the brain 23. Surgery followed by radiotherapy and chemotherapy is the most common modality of treatment. However, the prognosis of the condition remains poor, with a survival time of patients only 14 months 5. We conducted a retrospective study of 255 GBM patients from 2014 to 2016, of which 207 patients were followed up for more than 1 year. In these patients, the primary location of GBM were the frontal lobe in 84 cases, the temporal lobe in 67 cases, the parietal lobe in 35 cases, and the occipital lobe in 21 cases. We found that in all these cases, there was a degree of PTBE. Despite being commonly reported in the literature, the pathogenesis underlying GBM associated PTBE is unclear.

Currently international guidelines regarding the management of PTBE does not include a formal recommendation to surgically remove PTBE 24. In clinical practice, surgeons therefore use their clinical judgment to decide whether PTBE tissue should be resected. The standard surgical approach is to maximally resect the tumors while retaining maximum neurological function. We found no significant differences in postoperative complications between patients who received PTBE resection vs those who did not 25. The KPS scores were also not significantly different in patients with PTBE that was surgically managed compared to non-surgically managed patients 26. This suggests resection of PTBE tissue has no significant post-operative complications in patients with GBM.

Postoperative recurrence of tumor in patients with GBM is inevitable 27. In our study, all 207 patients relapsed within 6 months after surgery. However, when comparing recurrence between patients who received surgical resection of PTBE vs those who did not receive surgical treatment, we found that the relapse rate is delayed in the former group. This phenomenon has thus far not been reported in the literature. Interestingly, we also found that certain tumor associated proteins are abnormally expressed in the PTBE region. There was evidence of increased upregulation of AKT, ERK 1/2, PKM2, PGK1, LDHA, c-Myc and GLUT1 in the PTBE region 13,22,28,29. Meanwhile, tumor suppressor gene PTEN and P53 were down-regulated.

Various genes and molecular mediators have been shown to be upregulated in PTBE such as vascular endothelial growth factor (VEGF), aquaporins, Cox-2 and nitric oxide 30. However, in this study we have highlighted the presence of multiple other genes in PTBE tissue and we therefore speculate whether relapse of GBM is associated with pro-cancerous genes seen in the edematous regions.

## Conclusion

Our findings reveal that GBM is associated with PTBE and when this is surgically treated, can lead to a delay in relapse rates. We found no significant differences in neurological complications or KPS scores between the surgically and non-surgically managed groups. We found that PTBE tissue removal ameliorates the midline shift seen in GBM patients. We also found that postoperative recurrence of GBM patients is delayed in patients who received surgical treatment for PTBE. Most interestingly, we found increased expression of pro-tumor associated genes in the PTBE region, which may be associated with the relapse rate. Taken together, we show that GBM-induced PTBE may be a critical factor for postoperative recurrence in GBM patients and thus must be explored further to understand its importance in the pathobiology of GBM and its association with the tumor microenvironment.

## Figures and Tables

**Table 1:** Demographic characteristics of patients

GBM (patients)	255
**Age (years)**	
Median	52
Range	40-62
**Gender (patients)**	207
Male	114
Female	93
**Primary site of GBM (patients)**	207
Frontal lobe	84
Temporal lobe	67
Parietal lobe	35
Occipital lobe	21
**Edema on primary GBM (patients)**	163
Level 1	36
Level 2	127

**Table 2:** Postoperative symptoms

PTBE patients (163)							
	**Pre-operation**	**PTBE resection (85)**			**PTBE no resection (78)**		
		1 M	3 M	6 M	1 M	3 M	6 M
**Neurologic impairment**							
Headache	78	36	42	31	32	34	35
Vomiting	23	7	10	15	10	12	19
Paresthesia	43	26	29	33	28	30	34
Hemiplegia	56	26	33	39	30	36	38
Visual impairment	21	6	9	10	11	11	12
Aphasia	14	8	8	11	10	8	12
Seizures	12	4	3	5	6	4	5

**Table 3:** Postoperative complications

	Pre-operative	1M			3M			6M		
		PTBE resection (85)	PTBE no resection (78)	P value	PTBE resection (85)	PTBE no resection (78)	P value	PTBE resection (85)	PTBE no resection (78)	P value
KPS score										
≥70	149	81	76	0.468	76	73	0.341	79	71	0.652
<70	14	4	2	0.468	9	5	0.341	6	7	0.652
Midline shift										
<1 cm	63	76	60	0.032	58	42	0.059	40	23	0.214
≥1cm	100	9	18	0.032	27	36	0.059	45	55	0.214
Recurrence										
		0	2	0.227	10	28	0.004	74	48	0.0002
